# The relationship between academic self-efficacy and GenAI dependence among college students: a chain mediation model of academic stress and perceived usefulness of GenAI

**DOI:** 10.3389/fpsyg.2026.1784354

**Published:** 2026-07-10

**Authors:** Cai Zhang

**Affiliations:** School of Business, Jinling Institute of Technology, Nanjing, Jiangsu, China

**Keywords:** academic self-efficacy, academic stress, GenAI dependence, generative artificial intelligence, perceived usefulness of GenAI

## Abstract

**Background:**

As generative artificial intelligence (GenAI) becomes increasingly integrated into higher education, concerns have emerged regarding students' growing reliance on GenAI for academic tasks. Although prior studies have examined the educational benefits of GenAI, limited research has explored the psychological mechanisms underlying students' dependence on these technologies. This study investigated whether academic self-efficacy predicts GenAI dependence and examined the mediating roles of academic stress and perceived usefulness of GenAI.

**Methods:**

Using a time-lagged survey design, data were collected from 428 college students from five universities in eastern China. Participants completed validated measures of academic self-efficacy, academic stress, perceived usefulness of GenAI, and GenAI dependence. Structural equation modeling and bootstrap analyses were conducted to examine the hypothesized mediation pathways.

**Results:**

Academic self-efficacy was significantly and negatively associated with GenAI dependence. Academic stress and perceived usefulness of GenAI each served as significant mediators between academic self-efficacy and GenAI dependence. Furthermore, academic stress was positively associated with perceived usefulness of GenAI, and the sequential pathway from academic self-efficacy to academic stress, perceived usefulness of GenAI, and GenAI dependence was significant. Specifically, students with lower academic self-efficacy experienced greater academic stress, which increased their perceptions of the usefulness of GenAI and subsequently strengthened their dependence on GenAI.

**Conclusion:**

Academic self-efficacy affects college students' GenAI dependence both directly and indirectly through academic stress and perceived usefulness of GenAI. The findings extend the application of the I-PACE framework to the context of generative artificial intelligence and provide practical insights for universities seeking to promote responsible GenAI use, enhance students' independent learning capabilities, and mitigate excessive reliance on AI-assisted learning tools.

## 1 Introduction

Generative artificial intelligence (GenAI) has rapidly expanded its presence in higher education, transforming how college students engage with learning tasks such as information retrieval, writing, and problem solving (Zhang et al., 2024; Jia et al., 2025; Belkina et al., 2025; Lund, 2023). While GenAI offers substantial benefits in improving efficiency and accessibility (Lee and Low, 2024; Yusuf et al., 2024; Firat, 2023), its increasing integration into academic contexts has raised growing concerns about students' overreliance on these tools and the potential weakening of independent learning abilities (Robayo-Pinzon et al., 2025). In particular, the shift from using GenAI as an instrumental aid toward patterns of sustained reliance has emerged as an important issue for both educational research and institutional governance. However, despite the growing body of research on AI use in education, existing studies have primarily focused on usage frequency, learning outcomes, or ethical considerations, with limited attention to the psychological mechanisms underlying students' dependence on GenAI (Widyaningrum et al., 2024; Hu et al., 2023; Mhlanga, 2023). As a result, it remains unclear how individual characteristics, emotional experiences, and cognitive evaluations jointly contribute to the development of reliance on GenAI in academic contexts. Addressing this gap is essential for developing a more comprehensive understanding of technology-related dependence in the era of intelligent learning tools.

In recent years, increasing attention has been paid to various forms of AI-related reliance, including problematic AI use, AI overreliance, and cognitive offloading. Although these constructs are conceptually related, they capture different aspects of human–AI interaction. Problematic AI use generally refers to maladaptive or excessive engagement with AI systems that leads to negative psychological, behavioral, or functional outcomes, often resembling patterns of behavioral addiction (Hu et al., 2023). AI overreliance emphasizes the inappropriate delegation of tasks to AI systems, particularly in situations where independent judgment or critical thinking is required (Ray, 2023). Cognitive offloading, in contrast, describes a broader cognitive strategy in which individuals use external tools to reduce mental effort during information processing or problem solving (Sweller and Paas, 2017; Quintans-Júnior et al., 2023). In this study, GenAI dependence is conceptualized as a context-specific and relatively stable tendency toward psychological and behavioral reliance on generative artificial intelligence in academic tasks. This construct reflects dependence on the interactive technological system rather than on the specific content generated by the system (Zhang et al., 2024; Robayo-Pinzon et al., 2025; Chen et al., 2026). Unlike problematic AI use, it does not necessarily imply pathological symptoms. Compared with AI overreliance, it focuses less on decision accuracy and more on habitual reliance patterns. Relative to cognitive offloading, it emphasizes sustained reliance tendencies rather than temporary cognitive strategies. This distinction clarifies the conceptual boundaries of GenAI dependence and situates the present study within the broader literature on human–AI interaction and technology-related dependence. This conceptualization is also consistent with extensions of the I-PACE model. Which suggests that dependence-related behaviors may exist on a continuum ranging from non-problematic overreliance to more severe forms of behavioral addiction (Brand et al., 2016, 2019).

To better understand the psychological processes underlying such dependence, a theoretically grounded framework is needed to explain how individual traits, emotional responses, and cognitive evaluations interact to shape technology-related behaviors. The Interaction of Person–Affect–Cognition–Execution (I-PACE) model provides a comprehensive perspective for explaining the development of technology-related dependence by integrating personal factors, affective responses, and cognitive processes into a unified framework (Brand et al., 2019). According to the I-PACE model, individual characteristics influence behavioral outcomes through the sequential effects of emotional and cognitive mechanisms, forming a dynamic pathway of “individual characteristics → affective responses → cognitive appraisals → behavioral outcomes.” Within this framework, academic self-efficacy can be understood as a key personal factor reflecting students' beliefs in their academic capabilities (Bandura, 1997), academic stress as an affective factor capturing emotional responses to academic demands (Lazarus and Folkman, 1984), and perceived usefulness of GenAI as a cognitive factor representing evaluative judgments about the functional value of the technology (Davis, 1989). Together, these factors may jointly influence students' reliance on GenAI in academic contexts. In addition to the I-PACE model, cognitive load theory offers a complementary perspective for interpreting students' interactions with GenAI. From this viewpoint, students experiencing higher cognitive demands may rely more on external tools to reduce mental effort during learning processes (Sweller and Paas, 2017). In this study, cognitive load theory is incorporated as a supplementary interpretive perspective rather than a primary explanatory framework.

Accordingly, this study adopts the I-PACE model as the primary theoretical framework and incorporates cognitive load theory as a complementary interpretive perspective. It develops a structural equation model integrating academic self-efficacy, academic stress, perceived usefulness of GenAI, and GenAI dependence to systematically examine the formation mechanisms of GenAI dependence among college students in academic contexts. By distinguishing GenAI dependence from related constructs such as AI overreliance and cognitive offloading, and by integrating affective and cognitive pathways within an I-PACE-based framework, this study contributes to refining the conceptualization and mechanistic understanding of human–AI interaction in academic contexts.

## 2 Literature review and research hypotheses

### 2.1 Theoretical framework

#### 2.1.1 The I-PACE theory

The Interaction of Person–Affect–Cognition–Execution (I-PACE) model, proposed by Brand et al., is one of the most influential integrative frameworks for explaining digital technology dependence and related problematic use behaviors. The model posits that technology-related behavioral outcomes are not determined solely by technological features, but rather emerge from the dynamic interplay among personal characteristics, affective responses, cognitive appraisals, and executive processes (Brand et al., 2016, 2019; Ye et al., 2025). By emphasizing the sequential and interactive relationships among these components, the I-PACE model provides a comprehensive framework for understanding how dependence on emerging digital technologies develops over time. In particular, the I-PACE model highlights the mediating roles of affective states (e.g., stress) and cognitive evaluations (e.g., perceived usefulness) in linking individual characteristics to behavioral outcomes. This perspective has been widely applied to explain various forms of technology-related dependence (Hu et al., 2023; Rothen et al., 2018) and is therefore well suited to the present study, which focuses on dependence on generative artificial intelligence (GenAI) in academic contexts.

In this study, academic self-efficacy is conceptualized as the core personal factor, reflecting college students' relatively stable beliefs in their ability to successfully complete academic tasks. Academic stress represents the affective factor, capturing students' emotional responses to academic demands such as workload, time pressure, and performance expectations. Perceived usefulness of GenAI corresponds to the cognitive factor, reflecting students' evaluative judgments regarding the functional value of generative artificial intelligence in supporting learning activities. Finally, GenAI dependence represents the behavioral outcome, reflecting a shift from instrumental use to sustained reliance under the joint influence of personal, affective, and cognitive mechanisms. Based on this framework, the present study develops an analytical model of GenAI dependence that follows the pathway of “individual characteristics → affective responses → cognitive appraisals → behavioral outcomes.”

#### 2.1.2 Cognitive load theory as a complementary perspective

Cognitive Load Theory (CLT), originally proposed by Sweller, suggests that individuals' cognitive processing is constrained by the limited capacity of working memory. When task demands exceed available cognitive resources, learning effectiveness may be impaired, and individuals may seek external support to manage cognitive burden (Sweller, 1988, 2011; Kirschner et al., 2011). In the context of generative artificial intelligence (GenAI) in higher education, this perspective provides a useful complementary lens for interpreting students' interactions with AI tools. GenAI systems can assist with information processing, content generation, and task completion, thereby potentially reducing the cognitive demands associated with complex academic tasks (Kasneci et al., 2023; Daher and Hussein, 2024). As a result, students facing higher cognitive demands may be more inclined to rely on such tools as a means of alleviating perceived mental effort.

In the present study, CLT is not directly operationalized within the empirical model but is introduced as a supplementary interpretive perspective. It is used to help contextualize the relationship between academic stress, perceived capability constraints, and increased reliance on GenAI. Importantly, CLT is not treated as a competing explanatory framework but rather as a complementary perspective that aids in interpreting the observed patterns within the I-PACE-based model.

### 2.2 Research hypotheses

#### 2.2.1 The effect of academic self-efficacy on GenAI dependence

Academic self-efficacy refers to individuals' subjective judgments of their capability to successfully complete academic tasks and is a core construct underlying learning motivation and behavior (Honnicke and Broadbent, 2016; Li et al., 2020; Huang and Wu, 2025). Within the I-PACE framework, such stable individual-level characteristics are conceptualized as distal vulnerability or protective factors that can directly influence technology-related behavioral outcomes (Brand et al., 2019). In academic contexts, students with higher academic self-efficacy are more likely to engage in tasks independently and persist in the face of difficulty, relying on their own problem-solving strategies. In contrast, students with lower academic self-efficacy are more likely to perceive a mismatch between task demands and their own capabilities, which may increase uncertainty and reduce confidence in independent task completion. Under such conditions, generative artificial intelligence (GenAI) may be perceived as a readily available external resource that can reduce task-related difficulty and uncertainty through immediate feedback and efficient task support. Over time, repeated reliance on such external support may increase the likelihood of developing a stable tendency toward GenAI dependence. While low academic self-efficacy does not inevitably lead to dependence, it may increase susceptibility to reliance on external technological assistance, particularly when alternative support mechanisms are limited (Alshater, 2022; Bandura, 1997). Accordingly, the following hypothesis is proposed:

**H1**: Academic self-efficacy is negatively associated with GenAI dependence among college students.

#### 2.2.2 The mediating role of academic stress in the relationship between academic self-efficacy and GenAI dependence

Academic stress refers to a persistent state of psychological tension experienced by students in response to academic demands, time pressure, and perceived discrepancies between task requirements and their own capabilities (Bedewy and Gabriel, 2015; Struthers et al., 2000; Rani et al., 2023). It is a common affective response within contemporary learning environments (Reddy et al., 2018). According to the I-PACE model, stable individual characteristics do not typically translate directly into behavioral outcomes but instead influence behavior through their effects on affective states in specific contexts (Brand et al., 2019). In this framework, academic self-efficacy, as a core personal factor, is expected to shape students' emotional responses to academic demands, particularly their levels of academic stress. Students with lower academic self-efficacy are more likely to perceive academic tasks as exceeding their capabilities, resulting in heightened perceptions of uncertainty and reduced perceived control. Such appraisals are associated with increased academic stress, especially under conditions of performance evaluation and time constraints (Lazarus and Folkman, 1984; Vantieghem and Van Houtte, 2015; Nielsen et al., 2018). In contrast, students with higher academic self-efficacy tend to experience lower levels of stress due to greater confidence in their ability to manage academic challenges. Within this affective context, academic stress may further influence students' behavioral tendencies. Elevated stress levels may increase the likelihood that students seek external support to cope with academic demands. Generative artificial intelligence (GenAI), as an accessible and efficient tool, may be used as a coping resource to reduce perceived task difficulty and emotional strain. Over time, such reliance may contribute to the development of a stable tendency toward GenAI dependence. Although academic stress alone does not inevitably lead to dependence, It may increase susceptibility to reliance on external technological assistance, particularly when alternative coping or support mechanisms are limited (Kirschner, 2002). Accordingly, the following hypotheses are proposed:

**H2a**: Academic self-efficacy is negatively associated with academic stress among college students.

**H2b**: Academic stress is positively associated with GenAI dependence.

**H2**: Academic stress mediates the relationship between academic self-efficacy and GenAI dependence.

#### 2.2.3 The mediating role of perceived usefulness of GenAI in the relationship between academic self-efficacy and GenAI dependence

Perceived usefulness of GenAI refers to individuals' subjective evaluations of the extent to which generative artificial intelligence enhances learning efficiency and outcomes. It represents a central cognitive factor influencing both continued technology use and patterns of engagement (Choung et al., 2023; Davis, 2001). Within the I-PACE framework, academic self-efficacy, as a core personal factor, is expected to shape not only affective responses but also cognitive appraisals of external technological resources. In particular, individuals' beliefs about their own capabilities may influence how they evaluate the functional value of tools such as GenAI (Brand et al., 2019; Shakib Kotamjani et al., 2023). Students with lower academic self-efficacy are more likely to perceive a discrepancy between task demands and their own capabilities, which may increase their reliance on external resources to support task completion. Under such conditions, GenAI may be evaluated as a highly useful tool due to its ability to provide immediate feedback, reduce perceived task difficulty, and facilitate task completion. In contrast, students with higher academic self-efficacy may rely more on their own capabilities and therefore place relatively less emphasis on the instrumental value of external tools. Over time, repeated reliance on GenAI as a useful support tool may reinforce its perceived value and contribute to the formation of habitual use patterns. This process may increase the likelihood of developing a stable tendency toward GenAI dependence. Although perceived usefulness does not inevitably lead to dependence, it may strengthen individuals' tendency to rely on GenAI, particularly when it is consistently evaluated as an effective and efficient learning aid (Pitafi et al., 2020; Davis, 2001). Accordingly, the following hypotheses are proposed:

**H3a**: Academic self-efficacy is negatively associated with perceived usefulness of GenAI.

**H3b**: Perceived usefulness of GenAI is positively associated with GenAI dependence.

**H3**: Perceived usefulness of GenAI mediates the relationship between academic self-efficacy and GenAI dependence.

#### 2.2.4 The chain mediating role of academic stress and perceived usefulness of GenAI

The I-PACE model emphasizes that technology dependence develops through a sequential process involving “individual characteristics → affective responses → cognitive appraisals → behavioral outcomes” (Brand et al., 2019). This framework suggests that affective states may shape subsequent cognitive evaluations, which in turn influence behavioral tendencies. In academic contexts, students with lower academic self-efficacy are more likely to experience elevated academic stress in response to task demands and performance pressure. Such stress may alter how students evaluate external resources. Specifically, under conditions of heightened stress and perceived difficulty, students may place greater value on tools that can help them manage task demands and reduce uncertainty. As a result, generative artificial intelligence (GenAI) may be perceived as more useful due to its ability to provide efficient support and facilitate task completion (Davis, 1989; Ray, 2023; Lin et al., 2021). In this sense, academic stress may function as an intermediate affective condition that influences students' cognitive appraisals of GenAI. Through this process, lower academic self-efficacy may lead to higher stress, which in turn increases the perceived usefulness of GenAI, thereby contributing to a greater tendency toward reliance. Although this sequential process does not imply that all students will develop dependence, it suggests a potential pathway through which affective and cognitive factors jointly shape technology use behaviors. Accordingly, the following hypotheses are proposed:

**H4a**: Academic stress is positively associated with perceived usefulness of GenAI.

**H4**: Academic stress and perceived usefulness of GenAI jointly exert a chain mediating effect between academic self-efficacy and GenAI dependence.

**Figure 1:**
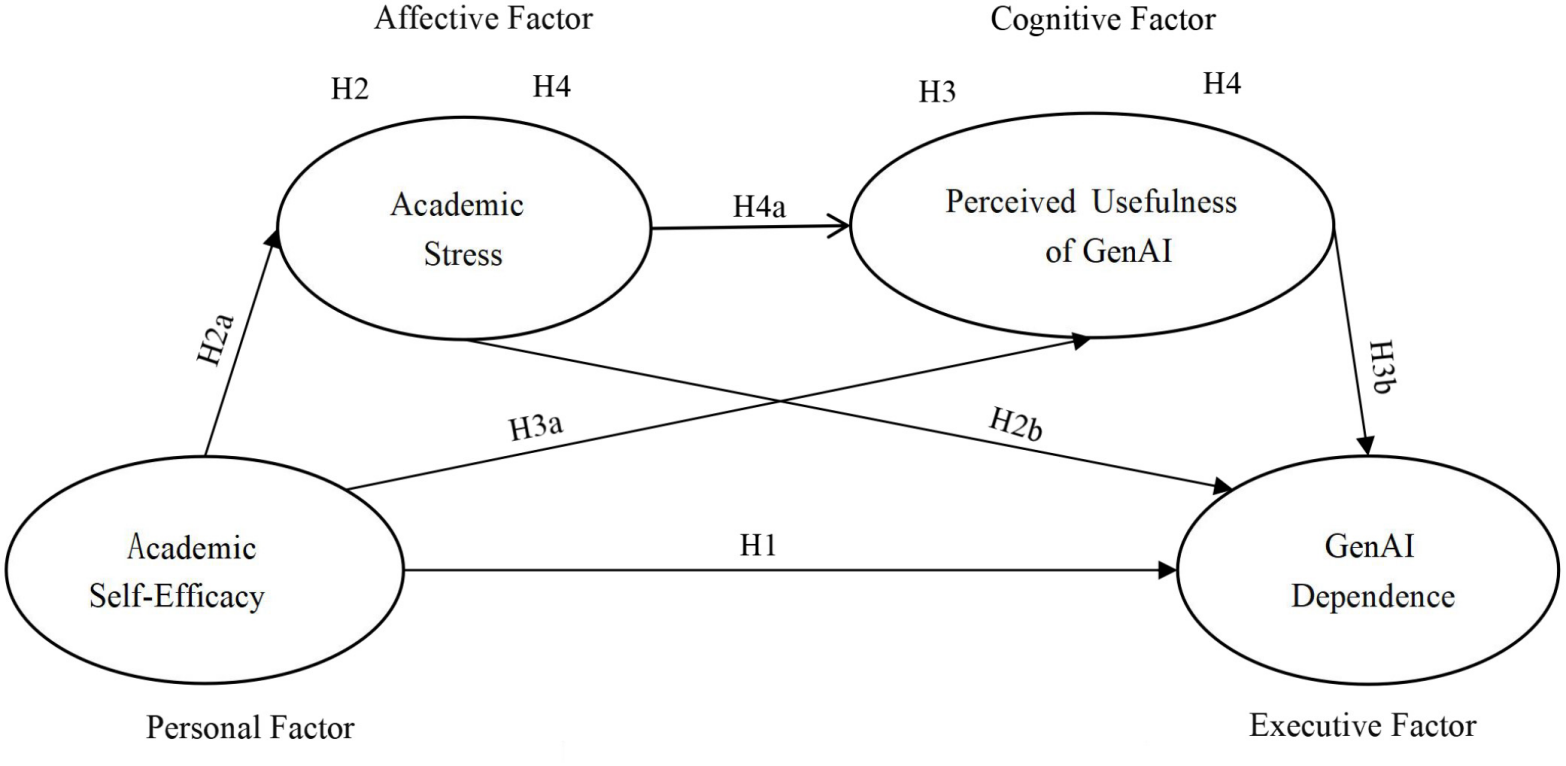
Research framework of study.

In summary, the research framework of the study is presented in Figure 1. Academic self-efficacy is conceptualized as the personal factor, academic stress as the affective factor, perceived usefulness of GenAI as the cognitive factor, and GenAI dependence as the executive outcome.

## 3 Research design

### 3.1 Ethics approval and informed consent

All methods were carried out in accordance with relevant guidelines and regulations. The study protocol was reviewed and approved by the Academic Ethics Committee of Jinling Institute of Technology. Informed consent was obtained from all participants prior to their participation in the study. All participants were informed that their responses would be used solely for academic research purposes and that their anonymity and confidentiality would be strictly protected.

### 3.2 Participants and procedure

This study conducted an online questionnaire survey between September and November 2025. Participants were recruited using a convenience sampling strategy from five universities located in eastern China. Recruitment was conducted through university-affiliated online communication channels, including class-based social media groups and student information networks. Participation was entirely voluntary, and students were invited to take part in a study examining experiences with generative artificial intelligence in academic contexts. The five participating institutions included both “Double First-Class” universities and regular undergraduate universities, providing variation in institutional characteristics and academic environments. However, because the sample was not randomly selected, the findings should not be interpreted as representative of all Chinese university students.

To encourage participation and reduce attrition across the two survey waves, respondents who completed both questionnaires received a small online monetary incentive equivalent to approximately RMB 5–10. The incentive was provided as compensation for participants' time rather than as a reward contingent upon specific responses. No identifying information other than the last four digits of participants' mobile phone numbers was collected for response matching purposes. A time-lagged data collection design was adopted, in which relevant data were collected in two stages separated by a three-week interval.

Specifically, during the first stage, Participants completed questionnaires containing demographic information and measures of academic self-efficacy and academic stress. A total of 500 questionnaires were distributed to students from five universities, and 459 valid responses were collected in this stage. In the second stage, the same 459 students were invited to complete questionnaires assessing perceived usefulness of GenAI and GenAI dependence. A total of 428 valid questionnaires were returned, yielding an effective response rate of 85.6%. Among the respondents, 196 were female and 232 were male. The sample included 85 freshmen, 116 sophomores, 144 juniors, and 83 seniors. The sample consisted of 236 students from regular undergraduate institutions and 192 students from “Double First-Class” universities. With respect to academic majors, 154 students were from humanities and social sciences, 150 from science and engineering disciplines, and 124 from other fields. Overall, the sample demonstrated a relatively balanced distribution in terms of gender, academic year, institutional type, and disciplinary category. These demographic variables were included in the analytical model as control variables to enhance the reliability and robustness of the research findings. The sample included students from multiple institutional types, the use of a non-probability sampling approach limits the extent to which the findings can be generalized to the broader population of Chinese university students.

Although detailed usage frequency was not directly measured, participants were recruited from university contexts where generative artificial intelligence tools are widely accessible and commonly used in academic tasks. During data collection, participants were informed that the questionnaire concerned experiences with GenAI in learning contexts, and all respondents confirmed familiarity with such tools before completing the survey. Therefore, the sample can be considered as comprising individuals with at least basic exposure to GenAI, providing a reasonable foundation for examining dependence-related tendencies.

### 3.3 Measurements

All measurement instruments used in this study were adapted from well-established scales in prior research. Minor modifications were made to ensure that the items were appropriate for the context of generative artificial intelligence (GenAI) use in higher education learning environments, thereby enhancing content validity. All items were measured using a five-point Likert scale ranging from 1 (strongly disagree) to 5 (strongly agree), with higher scores indicating higher levels of the corresponding constructs.

#### 3.3.1 Academic self-efficacy scale

Academic self-efficacy (AS) was measured using items adapted from the Academic self-efficacy scale developed by Nielsen et al. (2018) and Acosta-Enriquez et al. (2025). The scale consisted of 7 items (AS1~AS7) designed to assess students' confidence in their ability to successfully complete academic tasks independently. A sample item included: “I believe that I can achieve satisfactory academic performance through my own efforts.” The scale has also been widely applied in higher education research contexts, providing evidence of construct validity across diverse student populations (Hitches et al., 2022). In the present study, the academic self-efficacy scale demonstrated good internal consistency (Cronbach's α = 0.918).

#### 3.3.2 Academic stress scale

Academic stress (AST) was assessed using items adapted from the academic stress scale developed by Zhang et al. (2024). The scale included 5 items (AST1~AST5) measuring students' perceived pressure related to academic workload in learning contexts. A sample item included: “I often feel a high level of stress due to an excessive academic workload.” In the present study, the academic stress scale demonstrated good internal consistency (Cronbach's α = 0.899).

#### 3.3.3 Perceived usefulness of GenAI scale

Perceived usefulness of GenAI (AIPU) was measured using the scale developed by Choung et al. (2023). The scale included 5 items (AIPU1~AIPU5), assessing students' perceptions of the extent to which GenAI enhances learning efficiency and improves task performance. A sample item included: “Using GenAI tools helps me complete learning tasks more efficiently.” The perceived usefulness construct originates from the Technology Acceptance Model (Davis, 1989) and has been extensively validated in technology adoption research. Prior studies have consistently reported strong reliability (Cronbach's α = 0.920) and confirmed convergent and discriminant validity across diverse technological contexts (Choung et al., 2023). In the present study, the perceived usefulness scale demonstrated good internal reliability (Cronbach's α = 0.873).

#### 3.3.4 GenAI dependence scale

GenAI dependence (AID) was measured using the scale developed by Zhang et al. (2024). It should be noted that the scale captures students' tendency toward excessive reliance and psychological attachment to GenAI in academic contexts, rather than clinical-level dependence or addiction. The scale included 5 items (AID 1~AID 5) capturing students' excessive reliance on GenAI in academic activities. A sample item included: “I feel anxious when I am unable to use GenAI to complete learning tasks.” The GenAI dependence scale was adapted from Zhang et al. (2024), who reported satisfactory internal consistency (Cronbach's α = 0.852) and confirmed construct validity through structural equation modeling. The scale has been applied in studies examining problematic AI usage behaviors among university students (Sardi et al., 2025). In the present study, the GenAI dependence scale demonstrated good internal reliability (Cronbach's α = 0.906).

### 3.4 Data processing

This study employed SPSS 25.0 and AMOS 24.0 to conduct statistical analyses of the questionnaire data. SPSS 25.0 was primarily used for reliability and validity testing, as well as descriptive statistical and correlation analyses. AMOS 24.0 was used to conduct confirmatory factor analysis (CFA) and structural equation modeling (SEM) to assess model fit and test the hypothesized relationships.

## 4 Results

### 4.1 Common method bias test

Given that all variables in this study were measured using self-reported questionnaires, the potential issue of common method bias (CMB) was assessed. Following prior methodological recommendations, Harman's single-factor test was conducted to examine whether common method variance posed a serious threat to the validity of the results. The results indicated that four factors with eigenvalues greater than 1 were extracted. Importantly, the first unrotated factor accounted for 21.347% of the total variance, which is well below the commonly accepted threshold of 40% (Podsakoff et al., 2003). This result suggests that no single factor dominated the variance explanation and that common method bias was not a serious concern in the present study.

In addition to Harman's single-factor test, we conducted a common latent factor (CLF) analysis to further assess potential common method bias. Following established procedures, a latent method factor was added to the measurement model, with all observed indicators loading onto both their theoretical constructs and the common latent factor. The inclusion of the method factor did not substantially improve model fit (ΔCFI <0.01), and the standardized structural path coefficients remained largely unchanged (all changes <0.05). These results suggest that common method variance is unlikely to significantly bias the estimated relationships in the present study (Cheung and Rensvold, 2002; Williams et al., 1989). Therefore, the measurement instruments used in this research are unlikely to be substantially affected by common method bias and are considered appropriate for subsequent statistical analyses.

### 4.2 Reliability and validity tests

SPSS 25.0 was used to process the collected survey data and assess the internal consistency of each scale. The Cronbach's α coefficients were 0.918 for academic self-efficacy, 0.899 for academic stress, 0.873 for perceived usefulness of GenAI, and 0.906 for GenAI dependence. All Cronbach's α coefficients exceeded the recommended threshold of 0.80, indicating satisfactory internal consistency and suitability for subsequent analyses.

Validity reflects the extent to which measurement items accurately capture the intended constructs. Construct validity was assessed using the KMO measure and Bartlett's test of sphericity. In this study, the KMO value was 0.919, exceeding the recommended criterion of 0.90 and indicating excellent sampling adequacy. Bartlett's test of sphericity was significant (χ^2^ = 6302.161, *p* < 0.001), indicating that the data were suitable for factor analysis.

### 4.3 Confirmatory factor analysis

Confirmatory factor analysis (CFA) was conducted to further examine the reliability and validity of the measurement model. The results (see Table 1) showed that all standardized factor loadings exceeded 0.600. All composite reliability (CR) values exceeded the recommended threshold of 0.700. In addition, all average variance extracted (AVE) values were greater than 0.500. Together, the standardized factor loadings, CR values, and AVE values met the established criteria for convergent validity (Fornell and Larcker, 1981). Subsequently, Pearson correlation analysis was conducted, and discriminant validity was assessed by comparing the square roots of AVE values with the inter-construct correlation coefficients (see Table 1). The results showed that the square roots of the AVE values for all constructs were greater than their correlations with other variables, indicating satisfactory discriminant validity (Fornell and Larcker, 1981). Therefore, the measurement instruments used in this study met the standard requirements for reliability and validity.

**Table 1:** confirmatory factor analysis and correlation analysis results (*N* = 428).

Items	Outer loadings	AVE	CR	Constructs	AS	AST	AIPU	AID
AS1	0.847	0.612	0.916	AS	**0.782**			
AS2	0.633							
AS3	0.911							
AS4	0.905							
AS5	0.681							
AS6	0.735							
AS7	0.716							
AST1	0.812	0.642	0.900	AST	−0.397^**^	**0.801**		
AST2	0.779							
AST3	0.827							
AST4	0.821							
AST5	0.766							
AIPU1	0.802	0.582	0.874	AIPU	−0.456^**^	0.448^**^	**0.763**	
AIPU2	0.744							
AIPU3	0.829							
AIPU4	0.686							
AIPU5	0.744							
AID1	0.756	0.659	0.906	AID	−0.422^**^	0.411^**^	0.399^**^	**0.812**
AID2	0.786							
AID3	0.827							
AID4	0.832							
AID5	0.853							
				Mean	2.804	3.468	3.401	3.315
				SD	0.979	0.991	0.975	0.855

AS denotes academic self-efficacy, AST denotes academic stress, AIPU denotes perceived usefulness of GenAI, and AID denotes GenAI dependence. ^**^ indicates *p* < 0.01. The bolded values on the diagonal represent the square roots of the AVE values.

In addition, as shown in Table 1, significant correlations were observed among academic self-efficacy (Mean = 2.804, SD = 0.979), academic stress (Mean = 3.468, SD = 0.991), perceived usefulness of GenAI (Mean = 3.401, SD = 0.975), and GenAI dependence (Mean = 3.315, SD = 0.855). Academic self-efficacy was significantly negatively correlated with academic stress (*r* = −0.397, *p* < 0.01), perceived usefulness of GenAI (*r* = −0.456, *p* < 0.01), and GenAI dependence (*r* = −0.422, *p* < 0.01). Academic stress was significantly positively correlated with perceived usefulness of GenAI (*r* = 0.448, *p* < 0.01) and GenAI dependence (*r* = 0.411, *p* < 0.01). In addition, perceived usefulness of GenAI was significantly positively correlated with GenAI dependence (*r* = 0.399, *p* < 0.01). Collectively, these correlation patterns provided preliminary empirical support for the hypothesized relationships and justified subsequent hypothesis testing using structural equation modeling.

### 4.4 Structural model results

#### 4.4.1 Model fit indices

As shown in Table 2, the overall model fit indices indicate an acceptable fit between the measurement model and the observed data. Specifically, CMIN/DF = 2.481, CFI = 0.931, TLI = 0.923, and RMSEA = 0.059 meet the commonly recommended thresholds (Hu and Bentler, 1999), suggesting a satisfactory level of model fit. The GFI (0.876) and NFI (0.890) values were slightly below the conventional benchmark of 0.900. However, these indices have been shown to be sensitive to sample size and model complexity and are therefore considered less robust indicators in contemporary SEM practice, particularly in models with moderate complexity and adequate complementary fit statistics. In contrast, incremental and absolute fit indices such as CFI, TLI, RMSEA, and RMSEA are generally regarded as more reliable criteria for evaluating model fit (Hu and Bentler, 1999). Taken together, although GFI and NFI did not reach the most stringent cutoff values, the overall pattern of model fit indices supports an acceptable fit of the measurement model.

**Table 2:** Goodness-of-fit indices.

Criteria	Estimated model	Threshold	Author	Decision
CMIN/DF	2.481	Between 1 and 3	Hayduk, 1987	Acceptable
RMR	0.051	<0.100	Fornell and Larcker, 1981	Acceptable
RMSEA	0.059	<0.080	Bagozzi and Yi, 1988	Acceptable
GFI	0.876	>0.900 (conventional)	Hu and Bentler, 1999	Approximately acceptable
PGFI	0.721	>0.500	Mulaik et al., 1989	Acceptable
NFI	0.890	>0.900	Mulaik et al., 1989	Approximately acceptable
IFI	0.932	>0.900	Bollen, 1989	Acceptable
TLI	0.923	>0.900	Salum et al., 2012	Acceptable
CFI	0.931	>0.900	Bagozzi and Yi, 1988	Acceptable

To further evaluate model robustness, alternative partial mediation models were also examined. The comparison results indicated that these alternative specifications did not demonstrate superior model fit and offered less theoretically coherent explanations of the relationships among academic self-efficacy, academic stress, perceived usefulness of GenAI, and GenAI dependence. Therefore, the proposed model was retained as the final model for subsequent analyses.

#### 4.4.2 Direct path model results

**Figure 2:**
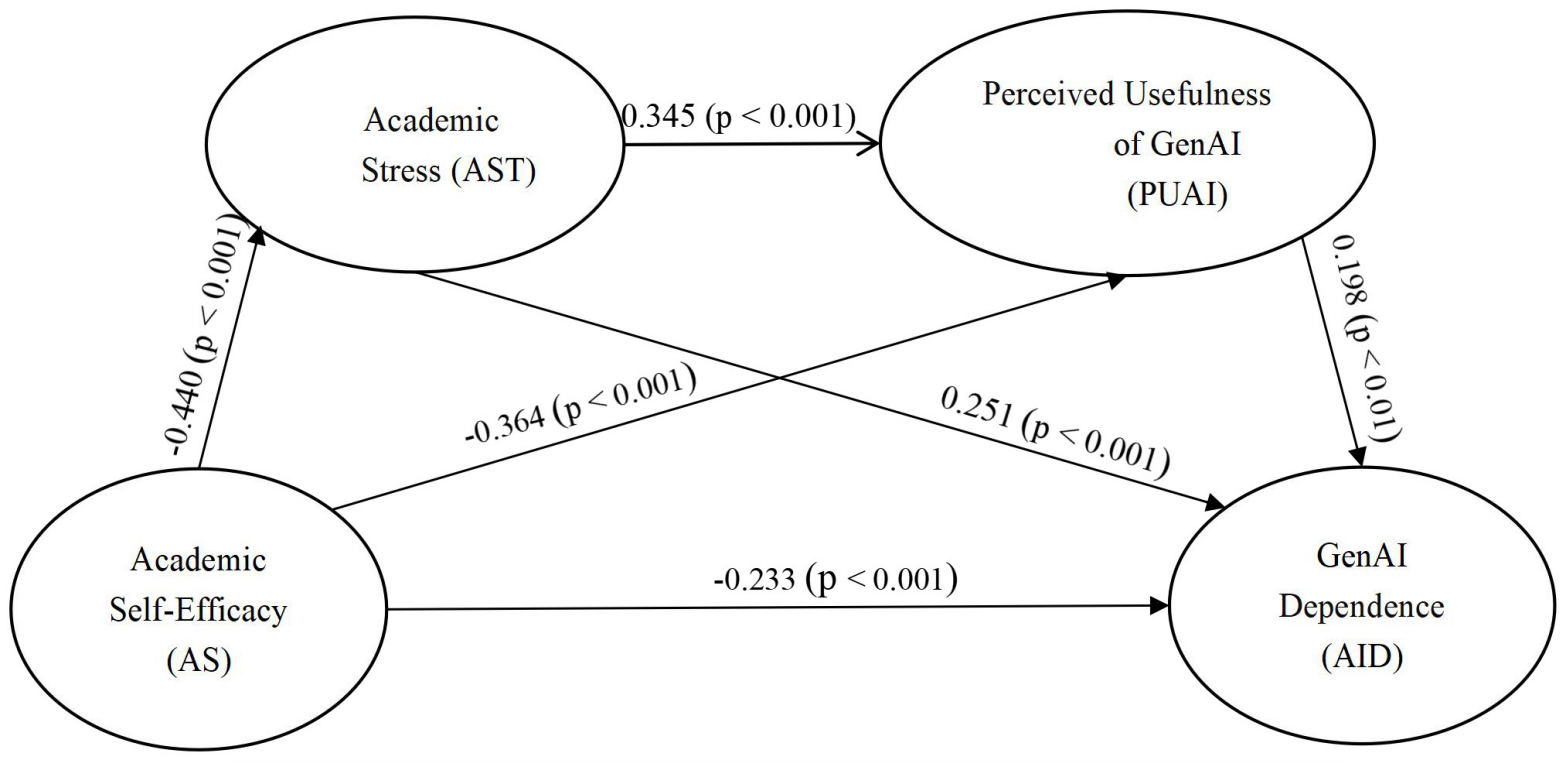
Representation of the model with the hypotheses.

Figure 2 and Table 3 summarize the results of the hypotheses evaluated in the model, with *p* < 0.05 indicating statistical significance. The results showed that academic self-efficacy was significantly negatively associated with GenAI dependence (β = −0.233, *p* < 0.001), thereby supporting Hypothesis H1. Academic self-efficacy was also significantly negatively associated with academic stress (β = −0.440, *p* < 0.001), supporting Hypothesis H2a. Academic stress was significantly positively associated with GenAI dependence (β = 0.251, *p* < 0.001), lending support to Hypothesis H2b. Academic self-efficacy was significantly negatively associated with perceived usefulness of GenAI (β = −0.364, *p* < 0.001), supporting Hypothesis H3a. In contrast, academic stress was significantly positively associated with perceived usefulness of GenAI (β = 0.345, *p* < 0.001), supporting Hypothesis H4a. Perceived usefulness of GenAI was significantly positively associated with GenAI dependence (β = 0.198, *p* < 0.01), supporting Hypothesis H3b. In summary, the structural model results provided empirical support for Hypotheses H1, H2a, H2b, H3a, H3b, and H4a.

**Table 3:** Direct path analysis.

Path relation	Standardized estimate	S.E.	C.R.	*P* value	Hypothesis
H1: academic self-efficacy → GenAI dependence	−0.233	0.041	−4.097	*P* < 0.001	Supported
H2a: academic self-efficacy → academic stress	−0.440	0.049	−8.477	*p* < 0.001	Supported
H2b: academic stress → GenAI dependence	0.251	0.045	4.311	*p* < 0.001	Supported
H3a: academic self-efficacy → perceived usefulness of GenAI	−0.364	0.051	−6.885	*p* < 0.001	Supported
H3b: perceived usefulness of GenAI → GenAI dependence	0.198	0.046	3.208	*p* < 0.01	Supported
H4a: academic stress → perceived usefulness of GenAI	0.345	0.055	6.397	*p* < 0.001	Supported
Gender → GenAI dependence	0.041	0.084	0.964	0.335	
Academic year → GenAI dependence	−0.08	0.041	−1.868	0.062	
Institutional type → GenAI dependence	−0.002	0.084	−0.043	0.966	
Disciplinary category → GenAI dependence	−0.02	0.052	−0.465	0.642	

In addition, gender, academic year, institutional type, and disciplinary category were included as control variables. None of these demographic variables showed statistically significant associations with GenAI dependence (all *p* > 0.05), indicating that the hypothesized relationships remained robust after accounting for demographic differences.

### 4.5 Mediation results

This study employed a bias-corrected nonparametric percentile bootstrap method with 5,000 resamples (*N* = 428) to test the mediating effects of academic stress and perceived usefulness of GenAI. The 95% confidence intervals of the indirect effects were estimated, and the results are presented in Table 4. In addition, this study interpreted the effect sizes of the standardized path coefficients (β*)* in accordance with Cohen (1988) criteria: *|*β*|* ≈ 0.10 indicates a small effect, *|*β*|* ≈ 0.30 indicates a medium effect, and |β| ≥ 0.50 indicates a large effect.

**Table 4:** Mediation effect analysis.

Effect type		Point estimate	Boot SE	Bias corrected 95% CI		Effect proportion
				Lower	Upper	
Mediating effect	H2: AS → AST → AID	−0.079	0.024	−0.133	−0.037	20.47%
	H3: AS → AIPU → AID	−0.052	0.019	−0.094	−0.018	13.47%
	H4: AS → AST → AIPU → AID	−0.022	0.009	−0.043	−0.008	5.70%
Direct effect	AS → AID	−0.233	0.056	−0.343	−0.120	60.36%
Total effect	AS → AID	−0.386	0.039	−0.404	−0.245	

AS denotes academic self-efficacy, AST denotes academic stress, AIPU denotes perceived usefulness of GenAI, and AID denotes GenAI dependence.

As shown in Table 4, for the mediating pathway AS → AST → AID, the Bootstrap 95% confidence interval was [−0.133, −0.037]. Since the confidence interval does not include 0, the mediating effect of AST is supported. The point estimate was −0.079, and the effect size falls within the small-effect range, indicating that although academic stress plays a significant mediating role in this pathway, its influence is relatively limited. Therefore, the original hypothesis H2 is supported.

For the mediating pathway AS → AIPU → AID, the Bootstrap 95% confidence interval was [−0.094, −0.018]. Since the confidence interval does not include 0, the mediating effect of AIPU is supported. The point estimate was −0.052, and the effect size likewise falls within the small-effect range, indicating that although perceived usefulness of GenAI can partially explain the formation of GenAI dependence, the strength of this pathway is relatively weak. Therefore, the original hypothesis H3 is supported.

For the mediating pathway AS → AST → AIPU → AID, the Bootstrap 95% confidence interval was [−0.043, −0.008]. Since the confidence interval does not include 0, the chain mediating effect of AST and AIPU is supported. The point estimate was −0.022, and the effect size also falls within the small-effect range. However, its statistical significance indicates that the progressive mechanism of “perceived ability deficiency → increased stress → enhanced tool value perception → dependence formation” genuinely exists. Therefore, the original hypothesis H4 is supported.

For the direct pathway AS → AID, the Bootstrap 95% confidence interval was [−0.343, −0.120]. Since the confidence interval does not include 0, the direct effect of AS is supported. The point estimate was −0.233, and the effect size falls within the small-to-moderate range. This indicates that although college students' academic self-efficacy can significantly reduce GenAI dependence, the strength of its influence is not substantial, suggesting that its effect may be manifested more prominently through mediating mechanisms. In summary, hypotheses H2, H3, and H4 were supported.

## 5 Discussion

Based on the I-PACE model as the primary analytical framework, this study further draws on cognitive load theory as a supplementary interpretive perspective. The role of cognitive load theory in this study is primarily interpretive rather than explanatory, and it is not intended to serve as a competing theoretical framework to the I-PACE model. This study systematically examines the psychological mechanisms underlying college students' GenAI dependence in academic contexts from an integrative perspective that considers academic self-efficacy, academic stress, and perceived usefulness of GenAI. Within the context of the present Chinese university sample, the findings indicate that students' reliance on GenAI does not simply stem from the technology's efficiency or accessibility. Rather, it is embedded in their self-evaluations of academic competence and the structure of academic stress they experience, the findings suggest a pattern in which lower perceived academic capability, higher academic stress, and stronger evaluations of GenAI usefulness are associated with greater levels of GenAI dependence. However, because the study was conducted exclusively among Chinese university students, these associations should be interpreted within the specific cultural and educational context in which they were observed rather than as universally applicable patterns.

Consistent with Hypothesis 1, academic self-efficacy exerted a significant negative effect on GenAI dependence. In academic contexts, among Chinese university students in the present sample, students with lower levels of academic self-efficacy tended to report greater reliance on generative artificial intelligence to complete learning tasks. This finding is consistent with previous research on digital technology dependence (Li et al., 2020; Parmaksiz, 2022; Odaci, 2011), suggesting that individuals with weaker confidence in their own abilities are more likely to seek external technological support. This result can be understood within the I-PACE framework, where academic self-efficacy functions as a personal factor influencing behavioral outcomes. Lower self-efficacy may increase individuals' reliance on external tools as a compensatory strategy, thereby reinforcing dependence-related behaviors. However, our findings differ from those of Zhang et al. (2024), who reported an indirect relationship between academic self-efficacy and AI dependency. In contrast, the present study identifies a direct negative association, which may be attributed to differences in measurement approaches, contextual factors, and the specific forms of AI use examined (i.e., general GenAI use versus ChatGPT-specific usage behaviors).

Furthermore, academic stress played a statistically significant mediating role in the relationship between academic self-efficacy and GenAI dependence. However, the magnitude of this indirect effect was relatively small (effect estimate = −0.079). Therefore, academic stress should be interpreted as one contributing factor rather than a dominant determinant of GenAI dependence. In practical educational settings, reducing academic stress may help mitigate reliance on GenAI, but substantial changes in dependence are unlikely to result from stress-reduction interventions alone. This finding is consistent with stress–coping theory (Lazarus and Folkman, 1984), which suggests that individuals under sustained stress may rely on external resources to manage emotional and task-related demands. It is also in line with the I-PACE framework, where negative emotional states can facilitate compensatory behaviors. From this perspective, higher levels of academic stress may increase students' tendency to rely on GenAI as a context-sensitive coping strategy under academic pressure. It should be noted that the strength and meaning of academic stress may vary across educational systems. Therefore, the observed relationship should be interpreted within the cultural and institutional context of Chinese higher education.

Additionally, perceived usefulness of GenAI also mediated the relationship between academic self-efficacy and GenAI dependence. This finding suggests that students' cognitive evaluations of the functional value of GenAI are associated with their reliance on these tools. Nevertheless, the indirect effect through perceived usefulness was relatively modest (effect estimate = −0.052), indicating that perceived usefulness explains only a limited portion of the overall relationship. From an educational perspective, interventions aimed at promoting more balanced perceptions of GenAI may contribute to responsible AI use, but they should be combined with broader educational strategies rather than viewed as standalone solutions. This finding is consistent with the Technology Acceptance Model (Davis, 1989), which identifies perceived usefulness as a key predictor of technology use. Students with lower academic self-efficacy may be more likely to perceive GenAI as an effective means of compensating for their perceived lack of ability. This perception may reinforce their evaluation of the technology's usefulness and, over time, contribute to increased reliance through repeated use. An alternative interpretation is that students with higher academic self-efficacy may engage with GenAI in qualitatively different ways. Rather than using it as a substitute for core cognitive effort, they may use it for refinement, idea expansion, or efficiency enhancement. In such cases, GenAI may be viewed as a supplementary tool rather than a necessary support, which may reduce the likelihood of developing dependence. From a cognitive perspective, these findings suggest that differences in self-efficacy may influence how students evaluate and utilize GenAI, which in turn shapes their behavioral tendencies. In addition, cognitive load theory provides a useful complementary lens for interpreting this pattern. Although cognitive load was not directly measured in this study, students with lower self-efficacy may experience greater perceived cognitive burden during learning tasks and may therefore rely more on GenAI as a form of cognitive offloading. However, this interpretation remains inferential and should be examined in future research. Furthermore, perceptions of the usefulness of GenAI may be influenced by broader cultural and educational contexts. For example, norms regarding technology adoption, expectations for independent learning, and institutional attitudes toward AI-assisted learning may shape how students evaluate the usefulness of GenAI. Therefore, the observed relationship between perceived usefulness and GenAI dependence should be interpreted primarily within the context of the present Chinese university sample, and its generalizability to other cultural settings remains to be established.

Finally, academic stress and perceived usefulness of GenAI also formed a chain mediating pathway between academic self-efficacy and GenAI dependence. Academic stress and perceived usefulness of GenAI formed a statistically significant chain mediating pathway between academic self-efficacy and GenAI dependence. However, the magnitude of this indirect effect was small (effect estimate = −0.022) and accounted for only 5.7% of the total effect. Therefore, this pathway should not be interpreted as a substantively decisive mechanism underlying GenAI dependence. Instead, it represents a modest but theoretically meaningful process through which affective and cognitive factors may jointly relate to students' reliance on GenAI. From an educational practice perspective, the findings suggest that interventions targeting stress management and students' perceptions of GenAI may produce incremental rather than transformative reductions in dependence. The practical value of these findings lies in identifying potential leverage points for supporting responsible AI use, rather than in demonstrating large behavioral effects. The results suggest a sequential process in which affective and cognitive factors jointly influence reliance on GenAI. Academic stress may function as an intermediate emotional condition that shapes students' cognitive evaluations of GenAI, leading them to attribute greater functional value to these tools under conditions of pressure. This process may, in turn, reinforce reliance behavior over time. Overall, these findings indicate that GenAI dependence is not solely driven by either emotional responses or rational evaluations, but may emerge through the interaction of affective and cognitive processes. In this sense, the development of dependence can be understood as a gradual process involving perceived ability limitations, stress responses, and reinforced cognitive evaluations.

Although the propose d model received empirical support, the observed relationships should not be interpreted as exhaustive explanations of GenAI dependence. Dependence may also be shaped by contextual and behavioral factors, such as AI literacy, disciplinary learning requirements, institutional AI governance policies, and prior digital-tool usage habits. These factors may operate alongside, or interact with, the psychological mechanisms identified in the present study. From a *post hoc* interpretive perspective, cognitive load theory provides a useful complementary lens for understanding the mechanism underlying these findings. Although cognitive load was not directly measured in this study, the results suggest that students with lower academic self-efficacy and higher academic stress may experience increased cognitive burden during learning processes. In such situations, generative artificial intelligence can function as a form of cognitive offloading, enabling students to reduce information-processing demands and task-related strain. Through repeated use, this cognitive offloading function may be associated with stronger reliance tendencies and higher levels of reported GenAI dependence. Importantly, this perspective complements, rather than replaces, the explanatory framework provided by the I-PACE model. Future research could incorporate direct measures of cognitive load to further examine this mechanism and strengthen the theoretical integration.

### 5.1 Theoretical and practical contributions

#### 5.1.1 Theoretical contributions

This study contributes to the literature in several ways. First, it extends the application of the I-PACE model to the context of generative artificial intelligence (GenAI) use in higher education. While the I-PACE framework has been widely used to explain addictive behaviors, its application to emerging academic technologies remains limited. By examining GenAI dependence among college students, this study demonstrates that the I-PACE model can also be used to understand non-clinical, context-specific forms of technology reliance. Second, this study contributes by examining the joint roles of affective and cognitive mechanisms in shaping GenAI dependence. Specifically, the findings show that academic stress and perceived usefulness function as mediating variables, suggesting that both emotional responses and cognitive evaluations are involved in the development of reliance on GenAI. This provides a more integrated perspective on how psychological processes are associated with technology use behaviors in academic contexts. Third, this study offers a process-oriented perspective on GenAI dependence by identifying a sequential pathway linking academic self-efficacy, academic stress, and perceived usefulness. Although the effect size of this pathway is relatively small, it highlights a possible mechanism through which individual perceptions, emotional responses, and cognitive evaluations may jointly influence behavioral tendencies. This perspective contributes to a more nuanced understanding of how technology reliance may develop over time.

#### 5.1.2 Practical implications

The findings of this study provide several practical implications for higher education institutions, educators, and students. First, the results suggest that students with lower academic self-efficacy are more likely to develop reliance on GenAI tools. Therefore, educators and institutions should focus on strengthening students' academic self-efficacy by providing appropriate learning support, scaffolding strategies, and feedback mechanisms. Enhancing students' confidence in their own abilities may reduce their tendency to rely excessively on external technological assistance. Second, given the role of academic stress in promoting GenAI dependence, it is important for universities to implement effective stress management interventions. This may include workload balancing, academic counseling services, and the development of supportive learning environments that reduce unnecessary pressure on students. Third, the findings highlight the importance of guiding students' perceptions of GenAI. Educators in similar educational contexts may consider to help students develop a more balanced understanding of GenAI as a supportive tool rather than a substitute for independent thinking. Instructional strategies could include promoting critical use of AI, encouraging reflective learning practices, and integrating AI literacy into curricula. Finally, Chinese universities may consider establishing guidelines for the appropriate use of GenAI in academic settings. Clear policies and educational initiatives can help prevent over-reliance while still allowing students to benefit from the advantages of these technologies.

It should also be noted that the mediating effects identified in this study were relatively small in magnitude. Consequently, the practical recommendations derived from these findings should be interpreted as supporting gradual improvements in students' AI-use behaviors rather than as interventions expected to generate immediate or substantial reductions in GenAI dependence. Sustainable changes are likely to require the combined influence of multiple educational, psychological, and institutional factors.

### 5.2 Limitations and future research

Although this study provides systematic evidence regarding the formation mechanisms of GenAI dependence, several limitations should be acknowledged. First, participants were recruited through a convenience sampling strategy from five universities in eastern China. Although the sample included students from different institutional types, including both “Double First-Class” and regular undergraduate universities, the non-probability sampling approach limits the generalizability of the findings. Future research could employ probability-based or stratified sampling methods to improve representativeness.

Second, this study did not include detailed measures of participants' actual usage of GenAI tools, such as frequency, duration, or types of use. Although participants were familiar with GenAI in academic contexts, the absence of fine-grained behavioral indicators may limit the extent to which dependence can be interpreted in relation to actual usage patterns. Future research should incorporate more detailed usage measures to strengthen the behavioral grounding of the construct.

Third, all participants were drawn from Chinese universities. Cultural factors related to academic competitiveness, examination-oriented education systems, and technology governance norms may shape students' perceptions of academic stress, self-efficacy beliefs, and attitudes toward GenAI tools. Therefore, the generalizability of the findings to other cultural contexts should be interpreted with caution. Future cross-cultural research could examine whether the proposed mechanism operates similarly in Western or other educational systems.

Fourth, although data were collected in two waves separated by a three-week interval, this design should not be interpreted as a full longitudinal design capable of establishing causal relationships. The temporal separation reduces some concerns regarding simultaneity, but the findings should still be interpreted as evidence of temporal associations rather than causal effects. Longitudinal designs with multiple waves or experimental manipulations of task complexity and GenAI access would provide stronger causal evidence.

Fifth, while cognitive load theory was used as a conceptual lens, no direct measures of intrinsic, extraneous, or germane cognitive load were included. As such, the cognitive offloading mechanism remains theoretically inferred rather than empirically verified. Future studies should incorporate validated cognitive load scales or experimental task manipulations to directly test CLT-derived predictions.

Sixth, the present study focuses primarily on psychological mechanisms derived from the I-PACE framework and does not explicitly examine several contextual and individual factors that may also contribute to GenAI dependence. For example, students with higher AI literacy may be more capable of using GenAI strategically without developing dependence, whereas students with lower AI literacy may rely more heavily on AI outputs. In addition, discipline-specific academic demands may shape the extent to which GenAI is integrated into learning activities. Institutional policies regarding AI use, as well as students' pre-existing habits of using digital learning tools, may also influence dependence tendencies independently of the psychological mechanisms examined in this study. Future research should investigate these factors as potential moderators or alternative explanatory variables to provide a more comprehensive understanding of GenAI dependence.

## 6 Conclusions

This study examined how college students develop dependence on generative artificial intelligence (GenAI) in academic contexts by the I-PACE framework and cognitive load theory as a supplementary interpretive perspective. Using structural equation modeling based on data from 428 university students, we identified a coherent psychological pathway linking academic self-efficacy, academic stress, perceived usefulness of GenAI, and GenAI dependence.

In the present sample of Chinese university students, academic self-efficacy emerged as a significant protective factor associated with lower levels of reported GenAI dependence. Students with stronger beliefs in their academic competence reported significantly lower levels of GenAI dependence. This suggests that reliance on generative AI is not merely a response to technological affordances, but is fundamentally shaped by individuals' perceived capability to independently manage academic tasks.

Academic stress emerged as a significant mediator between academic self-efficacy and GenAI dependence. Lower self-efficacy was associated with heightened academic stress, which was in turn associated with stronger dependence tendencies. This pattern implies that under sustained academic pressure, students may increasingly rely on GenAI as a compensatory mechanism to alleviate cognitive and emotional burden.

Perceived usefulness of GenAI also mediated the relationship between academic self-efficacy and dependence. Students with lower self-efficacy reported stronger perceptions of GenAI's instrumental value, and his heightened cognitive appraisal was significantly associated with dependence. This finding underscores the role of evaluative cognition in reinforcing repeated AI use and transforming functional engagement into stabilized reliance.

Importantly, the results were consistent with a sequential association involving academic self-efficacy, academic stress, perceived usefulness of GenAI, and GenAI dependence. Although the magnitude of this chain association was modest, it suggests that affective and cognitive factors may jointly relate to students' reported dependence on GenAI.

Taken together, these findings indicate that GenAI dependence among college students is associated with a combination of personal, affective, and cognitive factors. The observed relationships highlight the potential importance of academic self-efficacy, academic stress, and perceived usefulness in understanding patterns of reliance on GenAI within higher education contexts. Given that the study was conducted exclusively in Chinese higher education settings, the findings should be interpreted as context-dependent rather than universally applicable. Future cross-cultural studies are needed to examine the robustness of the proposed mechanism across different educational and cultural environments.

## Data Availability

The raw data supporting the conclusions of this article will be made available by the authors, without undue reservation.

## References

[B1] Acosta-Enriquez B. G. Ballesteros M. A. A. Guzman Valle M. A. Angaspilco J. E. M. Aquino Lalupú J. R. Jaico J. L. B. . (2025). The mediating role of academic stress, critical thinking and performance expectations in the influence of academic self-efficacy on AI dependence: case study in college students. Comp. Educ. Artif. Intell. 8:100381. doi: 10.1016/j.caeai.2025.100381

[B2] Alshater M. (2022). Exploring the role of artificial intelligence in enhancing academic performance: a case study of ChatGPT. SSRN Electron J. doi: 10.2139/ssrn.4312358

[B3] Bagozzi R. P. Yi Y. (1988). On the evaluation of structural equation models. J. Acad. Mark. Sci. 16, 74–94. doi: 10.1007/BF02723327

[B4] Bandura A. (1997). Self-efficacy: the Exercise of Control. New York, NY: W. H.Freeman.

[B5] Bedewy D. Gabriel A. (2015). Examining perceptions of academic stress and its sources among university students: the perception of academic stress scale. Health Psychol. Open 2:2055102915596714. doi: 10.1177/2055102915596714 28070363 PMC5193280

[B6] Belkina M. Daniel S. Nikolic S. Haque R. Lyden S. Neal P. . (2025). Implementing generative AI (GenAI) in higher education: a systematic review of case studies. Comput. Educ.: Artif. Intell. 8:100407. doi: 10.1016/j.caeai.2025.100407

[B7] Bollen K. A. (1989). A new incremental fit index for general structural equation models. Sociol. Methods Res. 17, 303–16. doi: 10.1177/0049124189017003004

[B8] Brand M. Wegmann E Stark R. Müller A. Wölfling K. Robbins T. W. (2019) The interaction of person-affect-cognition-execution (I-PACE) model for addictive behaviors: Update, generalization to addictive behaviors beyond internet-use disorders, specification of the process character of addictive behaviors. Neurosci. Biobehav. Rev. 104, 1–10. doi: 10.1016/j.neubiorev.2019.06.032 31247240

[B9] Brand M. Young K. S. Laier C. Wölfling K Potenza M. N. (2016). Integrating psychological and neurobiological considerations regarding the development and maintenance of specific internet-use disorders: an interaction of person–affect–cognition–execution (I-PACE) model. Neurosci. Biobehav. Rev. 71, 252–66. doi: 10.1016/j.neubiorev.2016.08.033 27590829

[B10] Chen J-Y. Xu T-Y. Tien T-Y. (2026). AI-mediated environments and the reconfiguration of symbolic convergence: cognitive implications for shared meaning formation. Front. Psychol. 17:1859863. doi: 10.3389/fpsyg.2026.1859863 42312048 PMC13269058

[B11] Cheung G. W. Rensvold R. B. (2002). Evaluating goodness-of-fit indexes for testing measurement invariance. Struct. Equ. Model. 9, 233–55. doi: 10.1207/S15328007SEM0902_5

[B12] Choung H. David P. Ross A. (2023). Trust in AI and its role in the acceptance of AI technologies. Int. J. Hum.–Comput. Interact. 39, 1727–39. doi: 10.1080/10447318.2022.2050543

[B13] Cohen J. (1988). Statistical Power Analysis for the Behavioral Sciences, 2nd Edn. New York, NY: Academic Press.

[B14] Daher W. Hussein A. (2024). Higher education students' perceptions of GenAI tools for learning. Information 15:416. doi: 10.3390/info15070416

[B15] Davis F. D. (1989). Perceived usefulness, perceived ease of use, and user acceptance of information technology. MIS Quart. 13, 319–40. doi: 10.2307/249008

[B16] Davis R. A. (2001). A cognitive–behavioral model of pathological Internet use. Comput. Hum. Behav. 17, 187–95. doi: 10.1016/S0747-5632(00)00041-8

[B17] Firat M. (2023). What ChatGPT means for universities: perceptions of scholars and students. J. Appl. Learn. Teach. 6, 57–63. doi: 10.37074/jalt.2023.6.1.22

[B18] Fornell C. Larcker D. F. (1981). Evaluating structural equation models with unobservable variables and measurement error. J. Mark. Res. 18, 39–50. doi: 10.1177/002224378101800104

[B19] Hayduk L. A. (1987). Structural Equation Modeling with LISREL: essentials and Advances. Baltimore, MD: Jhu Press. doi: 10.56021/9780801834783

[B20] Hitches E. Woodcock S. Ehrich J. (2022). Building self-efficacy without letting stress knock it down: stress and academic self-efficacy of university students. Int. J. Educ. Res. Open 3:100124. doi: 10.1016/j.ijedro.2022.100124

[B21] Honnicke T. Broadbent J. (2016). The influence of academic self-efficacy on academic performance: a systematic review. Educ. Res. Rev. 17, 63–84. doi: 10.1016/j.edurev.2015.11.002

[B22] Hu B. Mao Y. Kim K. J. (2023). How social anxiety leads to problematic use of conversational AI: the roles of loneliness, rumination, and mind perception. Comput. Hum. Behav. 145:107760. doi: 10.1016/j.chb.2023.107760

[B23] Hu L. T. Bentler P. M. (1999). Cutoff criteria for fit indexes in covariance structure analysis: conventional criteria versus new alternatives. Struct. Equ. Model. 6, 1–55. doi: 10.1080/10705519909540118

[B24] Huang T. Wu C. (2025). The chain mediating effect of academic anxiety and performance expectations between academic self-efficacy and generative AI reliance. Comput. Educ. Open 9:100275. doi: 10.1016/j.caeo.2025.100275

[B25] Jia W. Pan L. Neary S. (2025). Effect of GenAI dependency on university studentsâ academic achievement: the mediating role of self-efficacy and moderating role of perceived teacher caring. Behav. Sci. 15:1348. doi: 10.3390/bs15101348 41153138 PMC12561201

[B26] Kasneci E. Seßler K. Küchemann S. Bannert M. Dementieva D. Fischer F. . (2023). ChatGPT for good? On opportunities and challenges of large language models for education. Learn Individ. Diff. 103:102274. doi: 10.1016/j.lindif.2023.102274

[B27] Kirschner P. A. (2002). Cognitive load theory: implications of cognitive load theory on the design of learning. Learn. Instr. 12, 1–10. doi: 10.1016/S0959-4752(01)00014-7

[B28] Kirschner P. A. Ayres P. Chandler P. (2011). Contemporary cognitive load theory research: the good, the bad and the ugly. Comput. Hum. Behav. 27, 99–105. doi: 10.1016/j.chb.2010.06.025

[B29] Lazarus R. S. Folkman S. (1984). Stress, Appraisal, and Coping. New York, NY: Springer.

[B30] Lee C. C. Low M. Y. H. (2024). Using genAI in education: the case for critical thinking. Front. Artif. Intell. 7:1452131. doi: 10.3389/frai.2024.1452131 39554991 PMC11564148

[B31] Li L. Gao H. Xu Y. (2020). The mediating and buffering effect of academic self-efficacy on the relationship between smartphone addiction and academic procrastination. Comput. Educ. 159:104001. doi: 10.1016/j.compedu.2020.104001

[B32] Lin C. W. Lin Y. S. Liao C. C. Chen C. C. (2021). Utilizing technology acceptance model for influences of smartphone addiction on behavioural intention. Math. Probl. Eng. 2021:1–7. doi: 10.1155/2021/5592187

[B33] Lund B. D. (2023). Chatting about ChatGPT: how may AI and GPT impact academia and libraries. Libr. Hi Tech News 40, 26–9. doi: 10.1108/LHTN-01-2023-0009

[B34] Mhlanga D. (2023). Open AI in education: the responsible and ethical use of ChatGPT towards lifelong learning. Education doi: 10.2139/ssrn.4354422

[B35] Mulaik S. A. James L. R. Van Alstine J. Bennett N. Lind S. Stilwell C. D. (1989). Evaluation of goodness-of-fit indices for structural equation models. Psychol. Bullet. 105, 430–45. doi: 10.1037/0033-2909.105.3.430

[B36] Nielsen T. Dammeyer J. Vang M. L. Makransky G. (2018). Gender fairness in self-efficacy? A rasch-based validity study of the general academic self-efficacy scale (GASE). Scand. J. Educ. Res. 62, 664–81. doi: 10.1080/00313831.2017.1306796

[B37] Odaci H. (2011). Academic self-efficacy and academic procrastination as predictors of problematic internet use in university students. Comput. Educ. 57, 1109–13. doi: 10.1016/j.compedu.2011.01.005

[B38] Parmaksiz I. (2022). The mediating role of personality traits on the relationship between academic self-efficacy and digital addiction. Educ. Inf. Technol. 27, 8883–902. doi: 10.1007/s10639-022-10996-8

[B39] Pitafi A. H. Kanwal S. Khan A. N. (2020). Effects of perceived ease of use on SNS addiction through psychological dependence and habit: the moderating role of perceived usefulness. Int. J. Bus. Inf. Syst. 33, 383–407. doi: 10.1504/IJBIS.2020.105831

[B40] Podsakoff P. M. MacKenzie S. B. Lee J. Y. Podsakoff N. P. (2003). Common method biases in behavioral research: a critical review of the literature and recommended remedies. J. Appl. Psychol. 88:879. doi: 10.1037/0021-9010.88.5.879 14516251

[B41] Quintans-Júnior L. J. Gurgel R. Q. Araújo A. A. D. S. Correia D. Martins-Filho P. R. (2023). ChatGPT: the new panacea of the academic world. Rev. Soc. Bras. Med. Trop. 56, e0060–2023. doi: 10.1590/0037-8682-0060-2023 36888781 PMC9991106

[B42] Rani P. S. Rani K. R. Daram S. B. Angadi R. V. (2023). Is it feasible to reduce academic stress in net-zero energy buildings? Reaction from ChatGPT. Ann. Biomed. Eng. 51, 2654–6. doi: 10.1007/s10439-023-03286-y 37332007

[B43] Ray P. P. (2023). ChatGPT: a comprehensive review on background, applications, key challenges, bias, ethics, limitations and future scope. Internet of Things and Cyber-Phys. Syst. 3, 121–54. doi: 10.1016/j.iotcps.2023.04.003

[B44] Reddy K. J. Menon K. R. Thattil A. (2018). Academic stress and its sources among university students. Biomed. Pharmacol. J. 11, 531–7. doi: 10.13005/bpj/1404

[B45] Robayo-Pinzon O. Rojas-Berrio S. Camargo J. E. Foxall G. R. (2025). Generative artificial intelligence (GenAI) use and dependence: an approach from behavioral economics. Front. Pub. Health 13:1634121. doi: 10.3389/fpubh.2025.1634121 40843414 PMC12364812

[B46] Rothen S. Briefer J. F. Deleuze J. Karila L. Andreassen C. S. Achab S. . (2018). Disentangling the role of users' preferences and impulsivity traits in problematic Facebook use. PloS ONE 13:e0201971. doi: 10.1371/journal.pone.0201971 30183698 PMC6124717

[B47] Salum G. A. Patrick D. L. Isolan L. R. Manfro G. G. Fleck M. P. (2012). Youth quality of life instrument-research version (YQOL-R): psychometric properties in a community sample. J. Pediatr. 88, 443–8. doi: 10.2223/JPED.2193 22850664

[B48] Sardi J. Candra O. Yuliana D. F. Yanto D. T. P. Eliza F. (2025). How generative AI influences students' self-regulated learning and critical thinking skills? A systematic review. Int. J. Eng. Pedagogy 15, 94–108. doi: 10.3991/ijep.v15i1.53379

[B49] Shakib Kotamjani S. Shirinova S. Fahimirad M. (2023). “Lecturers perceptions of using artificial intelligence in tertiary education in uzbekistan,” in Proceedings of the 7th international conference on future networks and distributed systems (New York, NY: ACM), 570–8. doi: 10.1145/3644713.3644797

[B50] Struthers C. W. Perry R. P. Menec V. H. (2000). An examination of the relationship among academic stress, coping, motivation, and performance in college. Res. High. Educ. 41, 581–92. doi: 10.1023/A:1007094931292

[B51] Sweller J. (1988). Cognitive load during problem solving: effects on learning. Cogn. Sci. 12, 257–85. doi: 10.1207/s15516709cog1202_4

[B52] Sweller J. (2011). “Cognitive load theory,” in The Psychology of Learning and Motivation, eds. J. P. Mestre and B. H. Ross (Cambridge, MA: Academic Press), Vol. 55, 37–76 doi: 10.1016/B978-0-12-387691-1.00002-8

[B53] Sweller J. Paas F. (2017). Should self-regulated learning be integrated with cognitive load theory? A commentary. Learn. Instr. 51, 85–9. doi: 10.1016/j.learninstruc.2017.05.005

[B54] Vantieghem W. Van Houtte M. (2015). Are girls more resilient to gender-conformity pressure? The association between gender-conformity pressure and academic self-efficacy. Sex Roles 73, 1–15. doi: 10.1007/s11199-015-0509-6

[B55] Widyaningrum R. Wulandari F. Zainudin M. Athiyallah A. Rizqa M. (2024). Exploring the factors affecting ChatGPT acceptance among university students. Multidiscip. Sci. J. 6:e2024273. doi: 10.31893/multiscience.2024273

[B56] Williams L. J. Cote J. A. Buckley M. R. (1989). Lack of method variance in self-reported affect and perceptions at work: reality or artifact? J. Appl. Psychol. 74:462. doi: 10.1037/0021-9010.74.3.462

[B57] Ye J. H. Zhang M. Nong W. Wang L. Yang X. (2025). The relationship between inert thinking and ChatGPT dependence: an I-PACE model perspective. Educ. Inf. Technol. 30, 3885–909. doi: 10.1007/s10639-024-12966-8

[B58] Yusuf A. Pervin N. Román-González M. Noor N. M. (2024). Generative AI in education and research: a systematic mapping review. Rev. Educ. 12:e3489. doi: 10.1002/rev3.3489

[B59] Zhang S. Zhao X. Zhou T. Kim J. H. (2024). Do you have AI dependency? The roles of academic self-efficacy, academic stress, and performance expectations on problematic AI usage behavior. Int. J. Educ. Technol. High. Educ. 21:34. doi: 10.1186/s41239-024-00467-0

